# Re-emergence of syphilis in women of reproductive age and its association with the increase in congenital syphilis in Mexico during 2010–2019: an ecological study

**DOI:** 10.1186/s12879-021-06680-w

**Published:** 2021-09-23

**Authors:** Santa García-Cisneros, Antonia Herrera-Ortiz, Maria Olamendi-Portugal, Miguel Angel Sánchez-Alemán

**Affiliations:** Centro de Investigación sobre Enfermedades Infecciosas, Instituto Nacional de Salud Pública, Cerrada Los Pinos y Caminera, Cuernavaca, Universidad No. 655 Colonia Santa María Ahuacatitlán, Morelos, México

**Keywords:** Syphilis, Congenital syphilis, Sexually transmitted diseases, Mexico

## Abstract

**Background:**

Syphilis is a sexually transmitted infection that is re-emerging in different parts of the world. This infection can be transmitted during pregnancy, causing neonatal syphilis. The objective of this study was to examine trend in syphilis, congenital syphilis, and neonatal deaths among the Mexican population during 2010–2019.

**Methods:**

An ecological study was carried out to collect information about the incidence of syphilis, the incidence of congenital syphilis, and the incidence of neonatal death from congenital syphilis. The variables were described considering age, sex, Mexican state, and year. Trends across time (calendar year) were analyzed using linear regression, increases were estimated with 95% confidence intervals, and p < 0.05 was considered statistically significant.

**Results:**

The incidence of syphilis increased by an average of 0.336 cases/100,000 per year and was higher among women aged 15–19 years (0.693 cases). Cases of congenital syphilis increased from 62 cases in 2010 to 372 cases in 2019; furthermore, the increase in syphilis cases among women aged 20 to 24 years was associated with an increase in cases of congenital syphilis. In 2010, 50% of Mexican states did not report cases of congenital syphilis, but in 2018, only 10% did not report cases of congenital syphilis. Between 2010 and 2018, 83 neonatal deaths were reported, with the highest incidence in 2018 (0.88 deaths/100,000 newborns).

**Conclusion:**

The incidence of congenital syphilis is increasing in Mexico. As a consequence of the reemergence of syphilis among the population of reproductive age, it is necessary to address and treat syphilis in various population groups.

## Background

Syphilis is a sexually transmitted infection (STI) caused by the bacterium *Treponema pallidum subs pallidum*. Syphilis has four stages; the primary stage begins with a chancre, which appears on the glans, penis, labia majora, labia minora, perineum, or extragenital regions (palate, anus, rectus, fingers, or tongue) [1]. Secondary syphilis is characterized by systemic bacterial spread, erythematous rashes appearing on palms of hands, soles of feet, chest, and back. Later, there is a latency phase that lacks clinical manifestation, but nontreponemal serological tests remain positive; this stage can last up to 20 years. Infection remains continuous, and the tertiary stage develops, and this stage affects the cardiovascular, nervous, and musculoskeletal systems [1–3]. *T. pallidum* can cross the placental barrier, causing congenital syphilis. Approximately 50% of pregnant women infected with *T. pallidum* who are not treated transmit syphilis to their children before birth. In persons living with HIV, the natural history of syphilis is different: there are more patients with tertiary syphilis, patients are diagnosed earlier with Aids, and the presence of syphilis increases the transmission of HIV [4, 5].

According to the World Health Organization (WHO), in 2016, the worldwide incidence of curable STIs was estimated to be 376.4 million new cases among people aged 15 to 49 years; of these new cases, 6.3 million were syphilis, resulting in an incidence of 1.5 cases per 1000 inhabitants worldwide and 2.0 cases per 1000 inhabitants in America [6]. Furthermore, syphilis caused 200,000 stillbirths and deaths worldwide, making it one of the leading causes of newborn death. In the Americas, an estimated 22,800 cases of mother-to-child syphilis transmission were estimated, with a rate of 1.7 cases per 1000 live births [7]. In Mexico, an increase in the incidence of syphilis was observed between 2003 and 2013, especially among men aged 20–24 years and 25–44 years [8]. The objective of this study was to determine the trends of syphilis and congenital syphilis in Mexico between 2010 and 2019 through the information available in the General Directorate of Epidemiology of the Ministry of Health of Mexico.

## Methods

An ecological study was carried out to review the information about the incidence of syphilis, the incidence of congenital syphilis, and the number of cases of congenital syphilis from the Morbidity Yearbooks of the General Directorate of Epidemiology of the Ministry of Health of Mexico from 2010 to 2019. No administrative permissions were required to access the data; the information was freely accessible. The information about the incidences of syphilis and congenital syphilis was stratified by age, sex, year, and Mexican state, without any variable that could identify the participants. The number of neonatal deaths was obtained from the Epidemiological and Statistical Overview of Mortality due to Causes Subject to Epidemiological Surveillance in Mexico. From 2010 to 2018 (2019 is not yet available), the number of newborns for all years was obtained from the National Institute of Statistics and Geography of Mexico. In the present study, neonatal death was considered death in children under 1 year of age.

The analysis of the trends in syphilis incidence among women, syphilis incidence among men, congenital syphilis incidence, and syphilis incidence stratified by age group and sex from 2010 to 2019 was evaluated by measuring the average change by linear regression with 95% confidence intervals (95% CI). Linear regression was evaluated with standardized residual plots and R square to determine the curve of best fit (lineal, quadratic or exponential). Finally, comparisons were evaluated with Student’s t-test for slopes; p < 0.05 was considered statistically significant.

The number of cases of congenital syphilis was stratified into 0 cases, 1 case, 2–4 cases and 5 or more cases, considering the quartiles of the variable. A comparison, with Fisher’s test, was made between 2010 and 2019 considering each state of the republic. Subsequently, information on neonatal deaths from congenital syphilis was analyzed from 2010 to 2018, considering the number of cases and the number of newborns from each year to determine mortality.

Finally, the correlation between the trends in cases of congenital syphilis and incidence of syphilis in women aged 15–19 years and 20–24 years as well as the correlation between the incidence of congenital syphilis and the incidence of syphilis among women aged 20–24 years were evaluated for each state of the republic using the Spearman correlation test. The correlations were considered statistically significant when p < 0.05, and all the analyses were carried out with the statistical program GraphPad 10.0.

## Results

**Fig. 1:**
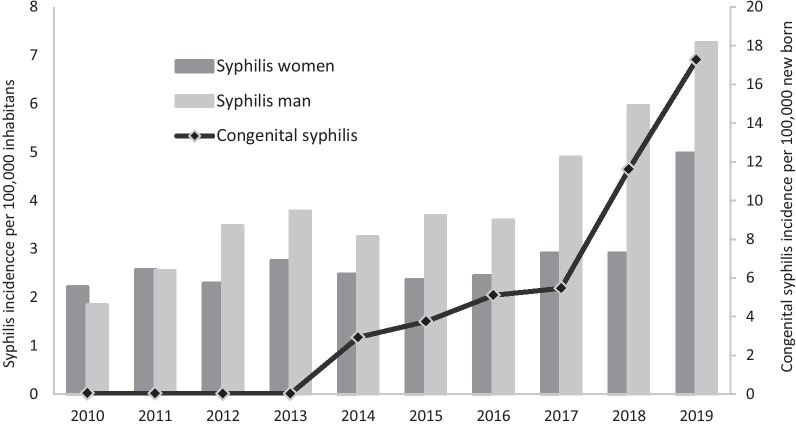
Syphilis incidence in Mexico, 2010–2019. The bars show the incidence of syphilis in Mexico between 2010 and 2019, stratified by sex. The line shows the incidence of congenital syphilis. In 2010, 2011, 2012 and 2013, the incidence of congenital syphilis was 0.05, 0.04, 0.02 and 0.02, respectively

The highest incidence of syphilis in the general population was observed in 2019, with 6.09 cases per 100,000 inhabitants, compared to 2.03 cases in 2010, with an average annual increase of 0.336 cases per 100,000 inhabitants (CI_95%_ 0.180–0.492, p = 0.001, r^2^ = 0.755). Since 2012, men have had a higher incidence of syphilis than women; the average increase among men was 0.482 cases per 100,000 inhabitants (CI_95%_ 0.303–0.660, p < 0.001, r^2^ = 0.829) compared to the increase among women, which was 0.178 cases per 100,000 inhabitants (CI_95%_ 0.017–0.339, p = 0.034, r^2^ = 0.448). The difference in the increases in male and female incidences was statistically significant (p = 0.010). An increase in congenital syphilis was observed, with a rate of 2.93 per 100,000 newborns in 2014 and up to 17.28 in 2019, with an average annual increase of 1.693 per 100,000 newborns (CI_95%_ 0.986–2.394, p < 0.001, r^2^ = 0.794), as shown in Fig. 1.

**Fig. 2:**
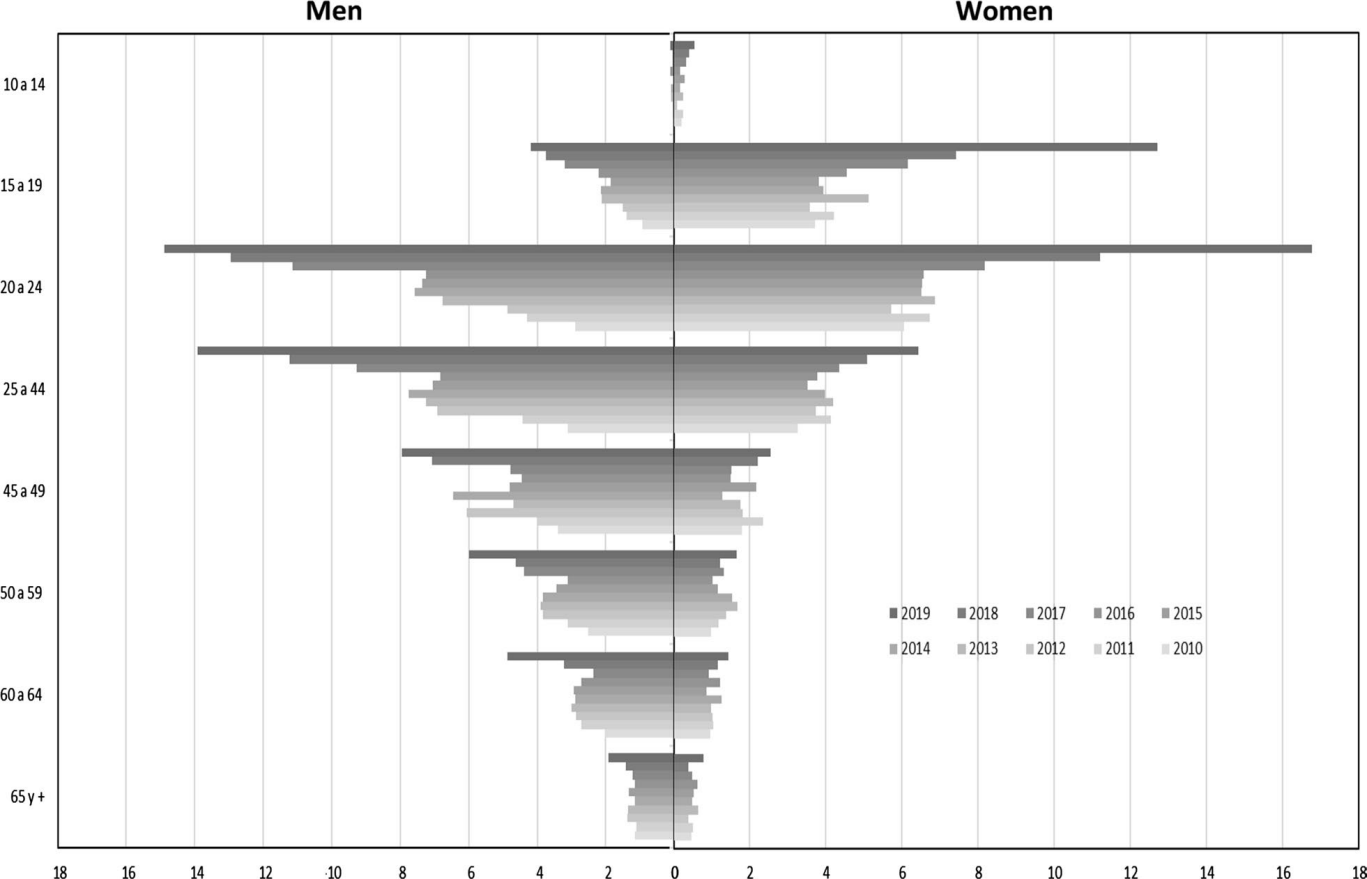
Syphilis incidence stratified by sex and age, Mexico 2010–2019. The figure shows the change in the incidence of syphilis by age group and sex from 2010 to 2019. The greater the intensity of the color, greater the calendar year

Among women, the highest incidence occurred in the 20–24-year-old age group, with 16.77 cases per 100,000 inhabitants in 2019 and an annual increase of 0.843 per 100,000 inhabitants (CI_95%_ 0.230–1.457; p = 0.013; r^2^ = 0.557), followed by the 15–19-year-old group, with an incidence of 12.7 cases per 100,000 inhabitants in 2019 and an average annual increase of 0.693 (CI_95%_ 0.191–1.194; p = 0.013; r^2^ = 0.560). Among men, the increase in incidence was highest in the 20–24-year-old group, with a rate of 14.89 cases per 100,000 inhabitants in 2019 and an annual increase of 1.080 per 100,000 inhabitants (CI_95%_ 0.654–1.505; p < 0.001, r^2^ = 0.810), followed by the 25–44-year-old group, with an incidence of 13.93 cases per 100,000 in 2019 and an annual increase of 0.991 cases per 100,000 (CI_95%_ 0.462–1.521, p = 0.002, r^2^ = 0.700), as shown in Fig. 2.

**Fig. 3:**
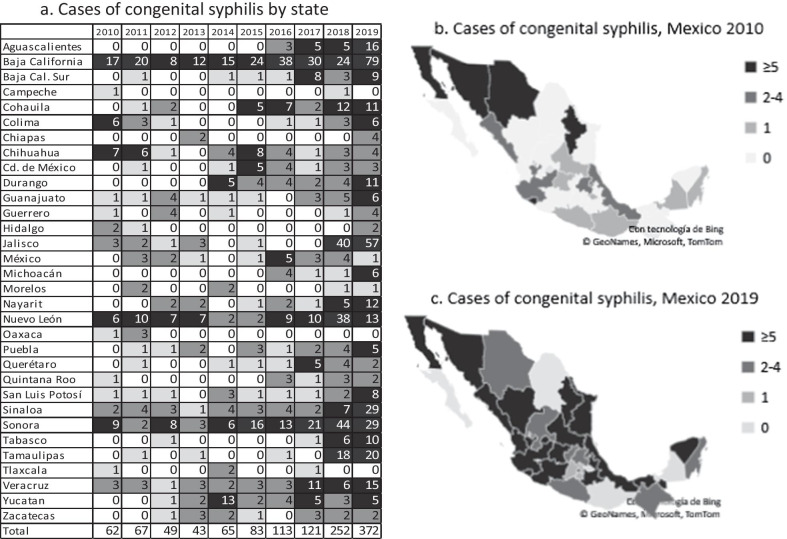
New cases of congenital syphilis by state, Mexico 2010–2019. **a** Shows the number of cases of congenital syphilis in each of the 32 Mexican states from 2010 to 2019. The darker colors indicate a greater number of cases. **b**, **c** Show the strained cases of congenital syphilis in 2010 and 2019 (with 0 cases, 1 case, 2–4 cases and ≥ 5 cases, considering the quartiles of the variable). A comparison with Fisher’s test, was made between 2010 and 2019. Microsoft product screen shots reprinted with permission from Microsoft Corporation

We reported a constant increase in new cases of congenital syphilis, with 62 cases in 2010, 83 cases in 2015, and 372 cases in 2019, demonstrating exponential growth (r^2^ = 0.747, p = 001). The states with the highest number of congenital syphilis cases in 2010 were Baja California, Colima, Chihuahua, Nuevo León, and Sonora, which reported between 6 and 17 cases, while in 2019, Baja California, Jalisco, Sinaloa, Sonora, and Tamaulipas reported between 20 and 79 cases of congenital syphilis. In addition, of the 32 states of Mexico, 16 did not report congenital syphilis cases in 2010; on the other hand, only three states did not report congenital syphilis cases in 2019, a difference which was statistically significant (p < 0.001). In 2010, only 5 states had 5 or more cases of congenital syphilis; in 2019, 19 states reported 5 or more cases of congenital syphilis (p < 0.001). The states of Campeche, Hidalgo, Morelos, and Tlaxcala had two or fewer congenital syphilis cases from 2010 to 2019, as shown in Fig. 3.

**Fig. 4:**
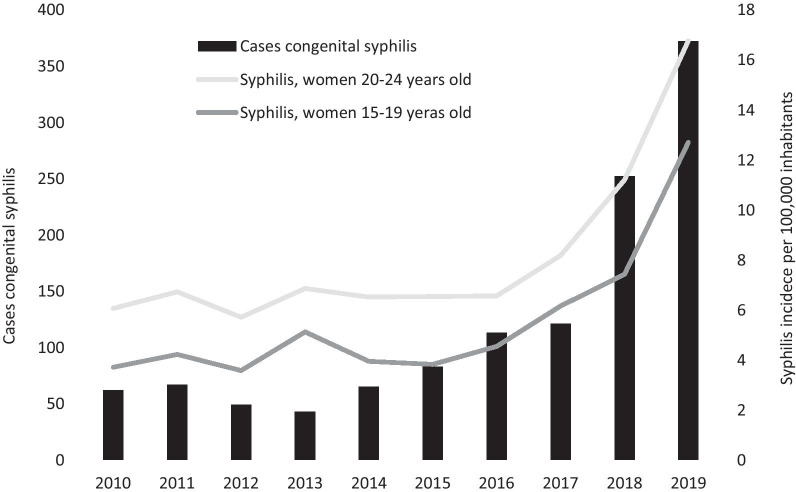
Syphilis among women aged 15–24 years and congenital syphilis, Mexico 2010–2019. The bars show the number of cases of congenital syphilis. The lines show the incidence of congenital syphilis in women aged 15–19 and 20–24 years, with r^2^ values of 0.904 and 0.958, respectively

**Fig. 5:**
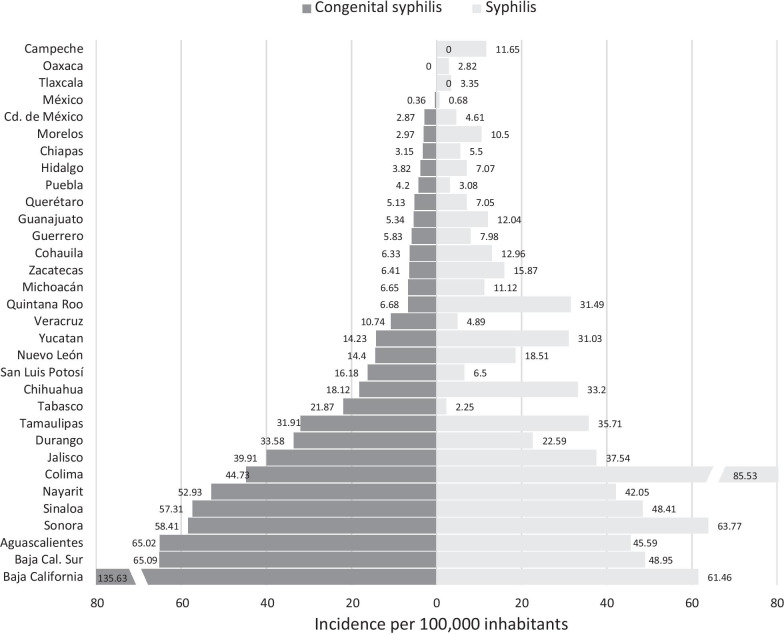
Syphilis among women aged 20–24 years and congenital syphilis, stratified by state of Mexico in 2019. The figure shows the relationship between the incidence of congenital syphilis and the incidence of syphilis in women in each of the Mexican states during 2019

The number of cases of congenital syphilis was correlated with the incidence of syphilis among women from 2010 to 2019 (r^2^ = 0.787, p < 0.001); however, this correlation was higher when stratifying by age. Among women aged 15–19 years, each increase in incidence unit showed an average increase of 36 new cases of congenital syphilis (slope 36.1, CI_95%_ 26.5–45.8, r^2^ = 0.904; p < 0.001); among women aged 20–24 years, this increase was an average of 30 cases per year (slope 30.4, CI_95%_ 24.8–36.0, r^2^ = 0.952; p < 0.001), as shown in Fig. 4. The correlation between syphilis and congenital syphilis by Mexican state was evaluated, and data of women aged 20–24 years from 2019 was used, which demonstrated an r^2^ = 0.621; p < 0.001. The seven states that reported an incidence of congenital syphilis greater than 40 cases per 100,000 live births were the same states that reported an incidence of syphilis greater than 40 cases per 100,000 women. States such as Morelos and Quintana Roo reported a higher incidence of syphilis and a lower incidence of congenital syphilis; in contrast, states such as San Luis Potosí, Tabasco and Veracruz reported a higher incidence of congenital syphilis and a lower incidence of acquired syphilis, as shown in Fig. 5.

The incidence of neonatal death due to congenital syphilis in children under 1 year of age in Mexico increased from 0.19 cases per 100,000 newborns (5 deaths) in 2010, 0.51 cases per 100,000 newborns (12 deaths) in 2015, and 0.88 cases per 100,000 newborns (19 deaths) in 2018. Information on other years was as follows: 2011 (0.39 cases and 10 deaths), 2012 (0.36 cases and 9 deaths), 2013 (0.28 cases and 7 deaths), 2014 (0.08 cases and 2 deaths), 2016 (0.35 cases and 8 deaths), and 2017 (0.49 cases and 11 deaths). Considering the total number of deaths between 2010 and 2018, the states of Jalisco (10), Baja California (9), Sonora (7) and Veracruz (6) reported the highest number of deaths; in contrast, the states of Guerrero, Hidalgo, Puebla, Quintana Roo, San Luis Potosí, Tlaxcala, Yucatán and Zacatecas did not report any neonatal deaths from congenital syphilis during the period analyzed.

## Discussion

According to the latest WHO estimates, the frequency of syphilis remained constant between 2012 and 2016, with a prevalence of 0.5%, in both men and women worldwide; however, in the Americas an increase from 0.7 to 0.9% was reported in both women and men [6]. The increase in syphilis has been documented among men who have sex with men and among people living with HIV in different parts of the world [9, 10]. In Mexico, an increase in the incidence of syphilis was reported among young men between 2003 and 2013, and it was hypothesized that they might be men who have sex with men as at that time, an increase in the incidence of syphilis among women as well as an increase in the incidence of congenital syphilis had not been reported [8]. With the results of the present study, we observed that the increase in the incidence of syphilis among men continues; however, we also observed an increase of more than double in the incidence of syphilis among women, as well as a great increase in the number of cases of congenital syphilis (6 times more cases).

The increase in the incidence of syphilis cases among women occurred mainly in those of reproductive age, leading to an increased risk of transmission of *T. pallidum* during pregnancy, which results in a greater number of cases of congenital syphilis and a greater number of neonatal deaths due to congenital syphilis [11]. In 2009, the Panamerican Health Organization proposed the elimination of maternal-child transmission of syphilis and HIV, later adding Chagas disease and hepatitis B [12–14]. One of the goals is to reduce the incidence of congenital syphilis to 0.5 or less per 1000 live births (0.005 per 100,000 live births). In the current study, a lower rate of congenital syphilis was observed; however, in 2019, the number of cases of congenital syphilis increased from 62 cases in 2010 to 372 cases, and the number of neonatal deaths increased from 5 in 2010 to 19 in 2018.

To reach the goal of reducing the incidence of congenital syphilis, one of the main recommendations is to promote 95% coverage of syphilis screening in pregnant women and 95% treatment [13]. However, this percentage of coverage is far from being achieved in Mexico; according to data from National Surveys (ENSANUT-2012 and ENSANUT-2018) [15, 16], only 43.6% of teenagers reported that they were tested for syphilis during their pregnancy, which increased to 56% in 2018, while among women aged 20–49 years, the number who were tested for syphilis increased from 39.9 to 62.5%. Increased testing for syphilis during pregnancy should lead to decreased numbers of cases of congenital syphilis; however, it is unknown whether women who test positive have access to treatment [14]. Syphilis screening during pregnancy is very important, in Mexico, it is mandatory to offer a syphilis test to pregnant women; however, changes and updates to the Official Mexican Standards, as well as the Clinical Guidelines, must be considered [17].

Young people also showed a greater increase in the incidence of syphilis; in women, the 15–24-year-old group had the highest rates, while among men, the 20–44-year-old groups had the highest rates. In the United States, it was reported that 45% of incident STI cases are concentrated in the population aged 15–24 years [18]. Sexual activity begins during this stage, and people this age may experiment with the use of illegal drugs; in addition, low condom use has been reported in this group. It is necessary to continue with the emphasis of STI prevention programs in this age group [19, 20].

There is great heterogeneity in the frequency of syphilis in the states of the Mexican Republic; some states have a directly proportional correlation between the frequencies of syphilis and congenital syphilis, and other states show no apparent correlation. Further research is needed to determine the reason for these differences, which may be at the biological level (immune status, circumcision, ectopy, coinfections), at the lifestyle level (number of sexual partners, concurrent partners, partner characteristics, condom use, sexual practices and seeking medical attention), at the population level (place of residence, access to services, prevention programs, quality of care, prevalence of STIs in the locality) or at the social structure level (social class, age, sex or ethnicity) [21–23]. All these variables possibly contribute to the great variation in the incidences of syphilis and congenital syphilis in Mexico, similar to that reported for herpes simplex virus type 2, which has a seroprevalence of 5.5% in the western region (Colima, Jalisco, Michoacán, and Nayarit) and a seroprevalence of up to 15.5% in the southwestern region (Chiapas, Guerrero and Oaxaca) [24].

A limitation of the present study includes not having more demographic, clinical or sexual behavior information. The definition of neonatal death was extended to children under 1 year of age, and information on stillbirth is lacking. In the case of women, it is unknown whether they were pregnant at the time of syphilis diagnosis; in addition, there is no information on the treatment of those who were diagnosed. There is the possibility of underreporting cases of syphilis, congenital syphilis, and neonatal deaths.

## Conclusions

There is an increase in the incidence of syphilis among different population groups that is most concentrated in the young population of reproductive age, which may cause an increase in the number of cases of congenital syphilis. It is necessary to strengthen different programs, such as early detection of syphilis, treatment and follow-up of diagnosed persons and trace of contacts, to help reduce this public health problem.

## Data Availability

Not applicable.

## Abbreviations

STI: Sexually transmitted infection
HIV: Human immunodeficiency virus
WHO: World Health Organization
95% CI: 95% Confidence intervals
